# Plasma CRP level is positively associated with the severity of COVID-19

**DOI:** 10.1186/s12941-020-00362-2

**Published:** 2020-05-15

**Authors:** Wei Chen, Kenneth I. Zheng, Saiduo Liu, Zhihan Yan, Chongyong Xu, Zengpei Qiao

**Affiliations:** 1 Department of Radiology, The Second Affiliated Hospital of Wenzhou Medical University, Yuying Chilldren’s Hospital, Wenzhou, China; 2 NAFLD Research Center, Department of Hepatology, The First Affiliated Hospital of Wenzhou Medical University, Wenzhou, China; 3 Department of Infectious Disease, The Sixth People’s Hospital of Wenzhou, Wenzhou, China; 4 Department of Laboratory Medicine, The Second Affiliated Hospital of Wenzhou Medical University, Yuying Children’s Hospital, No. 109 Western Xueyuan Road, Wenzhou, 325000 China

**Keywords:** SARS-CoV-2, Pneumonia, CRP, CT, Severity

## Abstract

**Aims:**

The coronavirus disease 2019 (COVID-19) is characterized as highly contagious and deadly; however there is no credible and convenient biomarker to predict the severity of the disease. The aim of the present study was to estimate whether the CRP level is able to act as a marker in indicating the severity of COVID-19.

**Methods:**

Patients who complained cough or chest pain with or without fever were enrolled after laboratory confirmed of SARS-CoV-2 viral nucleic acid via qRT-PCR. Chest computed tomography (CT) was then performed to classify the patients into mild, moderate and severe pneumonia groups according to the interim management guideline. Then linear regression models were applied to analyze the association between c-reactive protein (CRP) levels and severity of COVID-19 pneumonia.

**Results:**

When compared to mild pneumonia, the adjusted-Odds Ratio were 11.46, p = 0.029 and 23.40, p = 0.025 in moderate and severe pneumonia, respectively. The area under receiver operation curve was 0.898 (95% CI 0.835, 0.962, p < 0.001). Higher plasma CRP level indicated severe COVID-19 pneumonia and longer inpatients duration.

**Conclusions:**

The level of plasma CRP was positively correlated to the severity of COVID-19 pneumonia. Our findings could assist to discern patients of moderate to severe COVID-19 pneumonia from the mild ones. Our findings may be useful as an earlier indicator for severe illness and help physicians to stratify patients for intense care unit transfer.

## Background

Coronavirus belongs to the subfamily of *Ortho*-*coronavirinae* in the family of *Coronaviridae* and the *Order Nidovirales*. In 2003, a SARS-CoV had caused the outbreak of severe acute respiratory syndrome [1]. In December 2019, an “unknown viral pneumonia” outbreak has been reported. Finally, a novel coronavirus was detected, isolated virus was termed as SARS-CoV-2, characterized as highly contagious and deadly. By the end of February 2020, more than 78,631 cases infected with SARS-CoV-2, and more than 2747 deaths were confirmed in China, and the COVID-19 has been declared a pandemic by World Health Organization.

For those infected by SARS-CoV-2, some of the patients did not show hypoxemia or respiratory stress during the course of COVID-19, indicating a multifaceted disease of SARS-CoV-2 infection. Therefore, one reliable and convenient biomarker is needed to predict the severity of COVID-19 pneumonia. Recently, several studies have reported that C-reactive protein (CRP) is positively associated with severe dengue infection, and patients with higher plasma CRP in the initial period of dengue, are at higher risk to develop plasma leakage [2, 3].

We hypothesize that CRP could be utilized in predicting the severity of COVID-19 pneumonia. And to our knowledge, this is the first study to evaluate the prognostic ability of CRP in estimating the severity of COVID-19 pneumonia.

## Materials and methods

### Study design and population

Anonymous clinical data was collected and analyzed to facilitate better clinical decisions and treatment. The patients complained of cough, chest pain and other respiratory or digestive symptoms with or without fever, attending the Sixth People’s Hospital of Wenzhou, and the Second Affiliated Hospital of Wenzhou Medical University, were screened by qRT-PCR for SARS-CoV-2, and all the patients of positive results were included in this study. Upon admission, patients underwent blood routine test, chemical and immunological routine test, plasma CRP quantification, chest CT scanning to assess the severity of COVID-19, and treated with inhaled alpha interferon 2b and oral arbidol based on the latest management guideline by the Center for Diease Control and Prevention (CDCP) China [4]. Serial chest CT scanning were performed on every other day thereafter for monitoring disease progression and treatment effect. The patients are considered recovered and discharged when qRT-PCR is negative for three times at a 24 h interval. We assessed the severity of COVID-19 based on the combination of chest CT results, clinical assessments and laboratory findings. Diagnostic criteria for COVID-19 severity is based on the CDCP (China) Diagnosis and Treatment of COVID-19 [4].

### Sample collection

Oral swabs and venous blood samples were collected and examined by the central laboratory at Sixth Hospital of Wenzhou, and the Second Affiliated Hospital of Wenzhou Medical University, Yuying Children’s Hospital. For oral swabs, 1.5 mL DMEM and 2% FBS medium were added in each sterile tube. The supernatant was collected after a 2500 rpm centrifugation, 60 s vortex, and 15 to 30 s stewing. Then the supernatant was moved to lysis buffer for total RNA extraction. For each patient, vein blood samples were collected in two tubes, one anticoagulated with EDTA-K_2_ for blood routine test, and the other collected in vacant tube for serologic test. Serum was separated after a centrifugation at 3500 rpm for 10 min, and detected immediately. We only presented the patients who were positive for viral nucleotide detection.

### Total RNA extraction and qRT-PCR

A ReadyPrep Prot RNA Extract Kit (Sol/Insol, BioRad) was used to extract total RNA from 200 μL of oral swab supernatant, following the manufacturers’ instructions as mentioned elsewhere. RNA was eluted in 50 μL of elution buffer and immediately used as the template for RT-PCR detecting SARS-CoV-2, which could be found in previous studies. Briefly, RNA template was added in the qPCR system using HiScript II One Step qRT-PCR SYBR Green Kit (Vazyme Biotech Co., Ltd). The 20 μL qPCR reaction system contained 10 μL 2× One Step SYBR Green Mix, 1 μL One Step SYBR Green Enzyme Mix, 0.4 μL 50× ROX Refernce Dye, 0.4 μL of each primer (10 μM) and 2 μL RNA. The following program was performed for amplification: 50 °C for 3 min, 95 °C for 30 s followed by 40 cycles consisted of 95 °C for 10 s, 60 °C for 30 s, and a default melting curve step in an ABI 7500 machine [5].

### Computerized tomography (CT) protocol and grading

Chest CT scans were performed with a single inspiratory phase in one commercial multi-detector CT scanner (Optima CT540, GE Healthcare, U.S.A.). Patients were instructed on breath-holding to minimize the motion artifacts. CT images were acquired by the protocol of tube voltage, 100–120 kVp; effective tube current, 110–250 mAs, detector collimation, 0.625 mm; slice thickness, 1 mm; slice interval, 0.8 mm. Based on the raw data, the CT images were reconstructed by iterative reconstruction technique if possible.

**Fig. 1:**
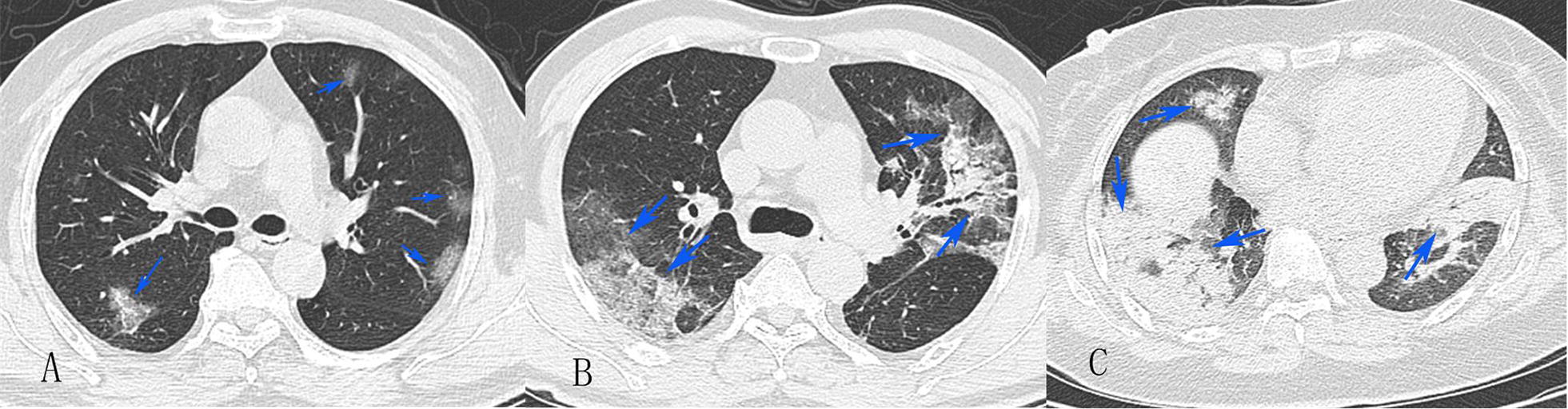
CT grading of COVID-19

The image analysis and grading were performed by three experienced radiologists, including Wei Chen, Zhihan Yan and Chongyong Xu, who have 10 to 15 years of experience in thoracic radiology, respectively. The final scores and grading were determined by consensus. The distribution of lung abnormalities was recorded as mild (axial CT shows peripheral and subpleural ground glass attenuation, Fig. 1a), moderate (the high-density shadow of plaques involving multiple lung lobes (≥ 3), CT shows ground glass, cloud flocculent or paving stone like changes, at least 2 lung lobes show pulmonary consolidation, local pulmonary fibrosis, and air bronchograms sign can be seen, Fig. 1b), or severe (CT showed diffuse consolidation [minimum of 80% of pulmonary or involving of 4 lobes] or cord like changes, and fibrosis was formed, Fig. 1c).

### Blood routine test and serologic test

Blood samples were analyzed by standard method in the central laboratory. Blood routine test was performed to count the blood cells and white blood cell classification, to measure C-reactive protein and hemoglobin concentration in the Mindray BC-5390 system (Shenzhen, China). The biochemical tests were performed in the VITROS 5600 Integrated Immunodiagnostic System (VITROS 5600, Johnson, New Jersey, U.S.A.), including albumin, total protein, lactic dehydrogenase, creatine kinase, alanine aminotransferase, aspartate transaminase, total bilirubin.

### Statistical analysis

Statistical data analysis was performed by using SPSS 25.0 (IBM, New York, U.S.A.). Data were presented as mean ± SD and percentages for continuous and categorical variables, respectively. The categorial variables were compared among the patients applying One-way analysis of variance (ANOVA), Kruskal–Wallis Chi square test, Fisher exact tests, and continuous variables were compared among the patients applying Students’ T test and ANOVA.

Multivariate linear regression model was applied to study the association between plasma CRP concentration with the severity of COIVD-19 pneumonia on chest CT performance. And receiver operation curve (ROC) was used to analyze the prognostic power of CRP level on the severity of COVID-19 pneumonia. The CRP cutoff value was calculated according to the largest Youden index: cutoff value = sensitivity − (1 − specificity).

## Results

### Baseline characteristics

Up to finishing of the paper, only 17 of the patients got recovered and released from hospital. All of the enrolled 76 patients infected with SARS-CoV-2 were confirmed by quantitative RT-PCR, on the first trial with throat swab. Nearly 55.3% (42 cases) of the patients were male, and there was no difference in sex ratio between CRP group 1 (< 20.44 mg/L) and CRP group 2 (≥ 20.44 mg/L). The average age of the patients was 44.5 years old, and the mean age was 41.9 years old and 49.1 years old in CRP 1 and CRP 2 respectively (p = 0.016). The highest mean body temperature of all patient during their hospital stay was 38.1 °C, whereas for the mean value of CRP 1 group was 38.0 °C while for CRP 2 group was 38.2 °C. Diabetes was not present in these patients. Prevalence of hypertension was 14.58% (7 cases) and 39.29% (11 cases) in the CRP 1 group and CRP 2 group, respectively. Cough, the most common respiratory symptom, were found in 64.68% (31 cases) and 67.86% (19 cases) in for CRP 1 and CRP 2 groups, respectively. Meanwhile, there were manifestations beyond respiratory system such as diarrhea and vomiting in both CRP 1 and CRP 2 groups. Nearly half of the patients in each group complained of fatigue (Table 1).

**Table 1:** Baseline characteristics of included patients, stratified by C-reactive protein

	CRP 1 (n = 48)	CRP 2 (n = 28)	*p* value
Demographics			
Age (years)	41.9 ± 13.3	49.1 ± 10.6	0.016
Male (%)	25 (52.1)	17 (60.7)	0.465
Highest temperature (degree centigrade)	38.0 ± 0.7	38.2 ± 0.6	0.223
Pulse per minute	92 ± 11	92 ± 12	0.786
Hypertension (%)	7 (14.58%)	11 (39.29%)	0.015
Respiratory manifestation			
Breath (times per minute) (%)			0.232
16	1 (2.080%)	0 (0.00%)	
17	1 (2.08%)	0 (0.00%)	
18	4 (8.33%)	1 (3.57%)	
19	3 (6.25%)	2 (7.14%)	
20	34 (70.83%)	19 (67.86%)	
21	3 (6.25%)	0 (0.00%)	
22	1 (2.08%)	5 (17.86%)	
23	1 (2.08%)	1 (3.57%)	
Dyspnea (%)	5 (10.42%)	9 (32.14%)	0.018
Cough (%)	31 (64.58%)	19 (67.86)	0.772
Manifestation of the other systems (%)			
Diarrhea	9 (18.75%)	8 (28.57%)	0.322
Vomitting	7 (14.58%)	6 (21.43%)	0.445
Fatigued	21 (43.75%)	16 (57.14%)	0.260
Laboratory characteristics			
Total protein (g/L)	70.88 ± 5.31	71.13 ± 6.55	0.854
Globulin (g/L)	28.70 ± 4.46	31.50 ± 5.24	0.016
Albumin globulin ratio	1.51 ± 0.26	1.29 ± 0.20	< 0.001
Lactic dehydrogenase (U/L)	203.21 ± 65.28	266.14 ± 83.33	< 0.001
Creatinine (mmol/L)	69.50 ± 15.02	73.32 ± 18.55	0.330
Alanine aminotranferase (U/L)	27.08 ± 26.47	35.21 ± 23.34	0.182
Aspartate transaminase (U/L)	28.00 ± 17.94	40.50 ± 25.66	0.015
Total bilirubin (mmol/L)	14.68 ± 8.18	15.12 ± 8.98	0.826
White blood cell count (10^9^/L)	4.57 ± 1.68	4.25 ± 1.10	0.377
Neutrophile count (10^9^/L)	2.90 ± 1.40	2.98 ± 0.82	0.806
Lymphocyte count (10^9^/L)	1.24 ± 0.55	0.94 ± 0.38	0.011
Neutrophile lymphocyte ratio	2.95 ± 3.05	3.77 ± 1.89	0.202
Hemoglobulin (g/L)	137.8 ± 12.5	134.3 ± 13.7	0.264
Platelet count (10^9^/L)	177.81 ± 68.38	171.64 ± 55.16	0.686

Data are presented as mean ± SD, or n (%). CRP 1: C-reactive protein < 20.44 g/L

CRP 2: C-reactive protein ≥ 20.44 g/L

The mean neutrophil count was 2.90 × 10^9^/L and 2.98 × 10^9^/L in CRP 1 and CRP 2 groups, respectively. The mean lymphocyte count was 1.13 × 10^9^/L in all enrolled patients, while it was 1.24 × 10^9^/L and 0.94 × 10^9^/L in CRP 1 and CRP 2 groups, respectively, (p = 0.011). The neutrophil lymphocyte ratio was 2.95 and 3.77 in CRP 1 and CRP 2 groups, respectively. The mean values of plasma globulin were 28.70 g/L and 31.50 g/L in CRP 1 and CRP 2, respectively (p = 0.016) (Table 1).

**Fig. 2:**
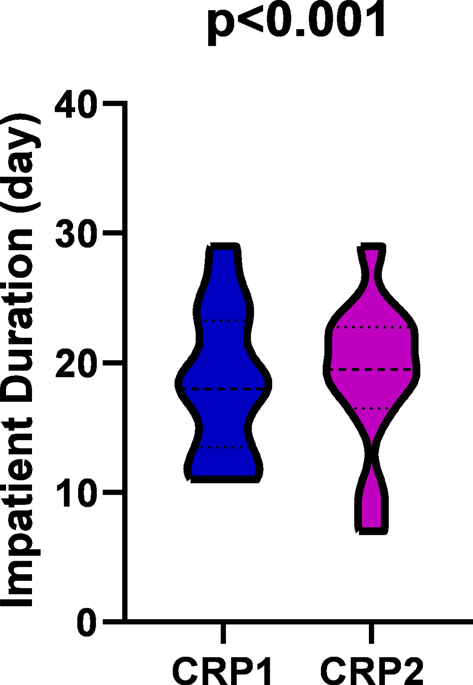
Higher CRP associated with longer inpatient duration

The inpatient duration time distribution was analyzed (Fig. 2), according to CRP stratification. It’s skewed in the CRP 2, in which the patients mostly experienced 19 to 22 days for treatment and recovery. However, in the CRP 1 group, the median inpatient time was 18 days from admission to recovery.

**Fig. 3:**
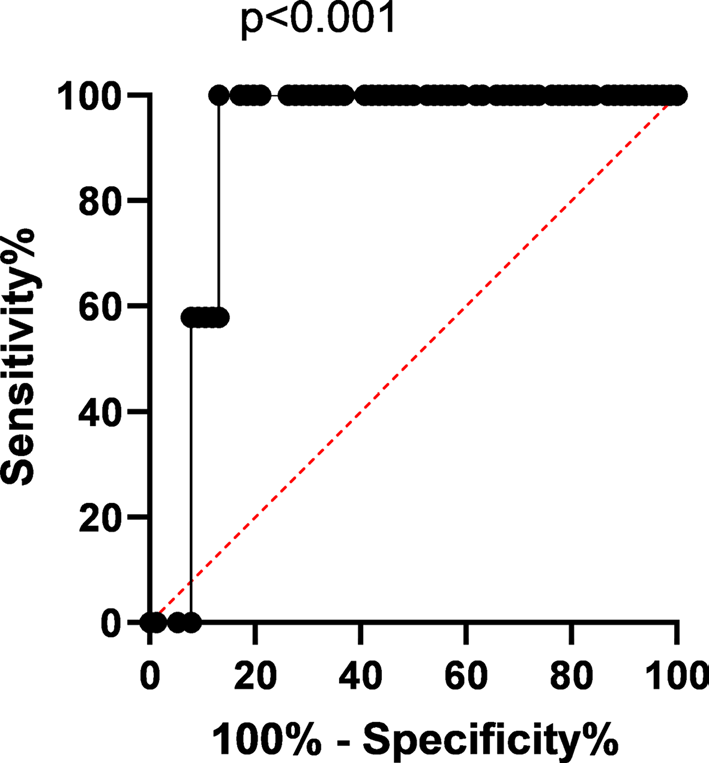
ROC curve of CRP diagnosing CT grading

The precise association between plasma CRP concentration and CT grading was analyzed with linear regression models (Table 2). When the CT grading increased from mild to moderate, the CRP concentration increased 11.47 mg/L (95% confidence interval 1.41, 21.52), p = 0.029; while when CT grading increased from mild to severe, CRP concentration increased 23.40 mg/L (95% CI 3.36, 43.45), *p* = 0.025, when adjusted for age, hypertension, lymphocyte count, albumin globulin ratio, aspartate transferase, and dyspnea. The ROC curve of CRP in diagnosing moderate-severe CT grading in the patient was analyzed (Fig. 3). The area under curve was 0.898 (95% CI 0.835, 0.962), *p* < 0.001.

**Table 2:** Relationship between CT grade with C-reactive protein

CT grading	C-reactive protein					
	Crude model		Adjusted model I		Adjusted model II	
	β (95% CI)	p value	β (95% CI)	p value	β (95% CI)	p value
1	0		0		0	
2	18.52 (9.32, 27.71)	< 0.001	11.64 (1.81, 21.46)	0.023	11.47 (1.41, 21.52)	0.029
3	46.83 (29.09, 64.57)	< 0.001	31.94 (13.16, 50.72)	0.001	23.40 (3.36, 43.45)	0.025

Crude Model adjusted for None

Adjusted Model I adjusted for age, lymphocyte count, albumin globulin ratio

Adjusted Model II adjusted for age, lymphocyte count, albumin globulin ratio, hypertension, aspartate transferase, dyspnea

## Discussion

Since the outbreak of SARS-CoV-2 in Wuhan city, China, it was characterized as highly contagious and deadly. Up to the end of February 2020, a total of over 78,631 cases of infections have been confirmed, with 2747 cases of death. This is the first study on the prognostic power of CRP for moderate-severe CT grading and inpatient duration in COVID-19 infected patients.

In the present retrospective observational study, a positive correlation between plasma CRP level and CT grading was found (Table 2), independent of age and lymphocyte count. When compared to the mild CT manifestation, the CRP concentration increased significantly by 11.47 mg/L and 23.40 mg/L, in the moderate and severe group respectively, *p* < 0.05. Furthermore, the diagnostic performance of CRP on CT grading was analyzed with ROC (Fig. 3) and compared with Student’s t test. The area under curve was 0.898, p < 0.001, indicating prominent diagnostic power on the COVID-19 progression on CT. Subsequently, our results yielded a discriminatory power of higher CRP levels in moderate-severe SARS-CoV-2 pneumonia patients from lower CRP levels in those with mild condition, with a cutoff level of 16.60 mg/L (77.0% sensitivity, 72.0% specificity). The corresponding sensitivities and specificities of plasma CRP level could be found in Table 3.

**Table 3:** The sensitivity and specificity of different CRP levels

Plasma CRP level (mg/L)	Sensitivity	1-specificity
0.55	0.02	0.00
0.70	0.09	0.00
1.10	0.14	0.00
1.50	0.16	0.00
1.65	0.18	0.00
1.80	0.21	0.00
2.05	0.23	0.00
2.30	0.27	0.03
2.65	0.29	0.03
3.00	0.32	0.03
3.35	0.34	0.03
3.70	0.39	0.09
4.40	0.41	0.09
5.05	0.41	0.13
5.65	0.43	0.13
6.45	0.46	0.13
6.80	0.48	0.13
7.10	0.50	0.13
7.50	0.52	0.13
7.80	0.52	0.16
8.45	0.55	0.22
9.75	0.57	0.22
10.75	0.57	0.25
11.05	0.59	0.25
11.55	0.61	0.25
12.10	0.64	0.25
12.35	0.66	0.25
13.10	0.68	0.25
13.90	0.71	0.28
14.35	0.73	0.28
15.25	0.75	0.28
16.60	0.77	0.28
18.20	0.77	0.31
19.25	0.79	0.31
20.65	0.82	0.38
21.75	0.84	0.41
22.05	0.86	0.41
22.30	0.86	0.44
23.50	0.89	0.47
24.35	0.91	0.47
24.95	0.91	0.50
25.85	0.91	0.56
26.60	0.91	0.59
31.15	0.93	0.59
36.55	0.95	0.59
38.10	0.98	0.59
39.00	0.98	0.63
40.60	0.98	0.66
42.20	0.98	0.72
43.75	0.98	0.75
44.90	0.98	0.78
47.85	1.00	0.78
51.75	1.00	0.81
58.10	1.00	0.84
69.10	1.00	0.88
84.70	1.00	0.91
98.20	1.00	0.94
104.05	1.00	0.97
107.20	1.00	1.00

Our results were supported by several studies about CRP level with disease severity elsewhere. In dengue’s infection, CRP has been suggested to be used as a prognostic marker, and higher levels of CRP indicating increased risk of disease progression [6, 7]. It’s noticeable that dengue virus and SARS-CoV-2 are RNA virus, sharing similarity in the course of infection. CRP is rapidly synthesized by hepatocytes when stimulated by inflammation. It binds to a variety number of eukaryotic and prokaryotic pathogens, facilitating complement activation through classical pathway [8], indicating immune activation, lymphocyte infiltration, immune molecules consumption and inflammation outbreak. Clinically, increased CRP levels might be early indicators of nosocomial infections in COVID-19 patients who were slow to recover, and might aid physicians to administer empirical antibiotics treatment early to prevent worsened outcome [9, 10].

**Fig. 4:**
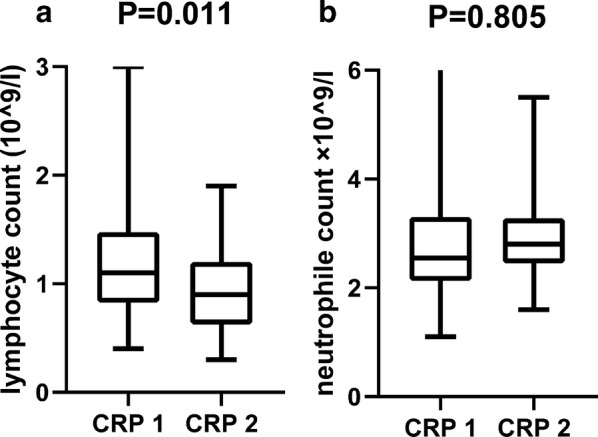
CRP association with lymphocyte and neutrophile

Though the overall mean value of circulating lymphocyte count was among the normal intervals for adults in China (95% CI 1.1 × 10^9^/L, 3.2 × 10^9^/L), it was found significantly lower in CRP 2 group (Fig. 4a) than CRP 1 group and normal intervals, indicating a quick lymphocyte infiltration and severe inflammation, which was in accordance with the findings mentioned above. However, there was no difference of neutrophil count in the two subgroups (Fig. 4b), indicating the innate immunity played less role in the early stage of SARS-CoV-2 pneumonia. Furthermore, the patients of CRP 2 group experienced significantly longer inpatient duration than that of CRP 1 group, which are reasonably explained by the more severe illness for patients in CRP 2 group.

The most important limitation of our study is the single ethnic participants, tending to selection bias. However, the SARS-CoV-2 is highly deadly and contagious, thus we can only enrolled those we can help. The other limitation is relatively small observation cohort. This effect could be minimized by further exploration in future larger observational study.

## Conclusion

The plasma CRP level is positively correlated to the severity of COVID-19 on CT performance, and higher level of CRP showed a longer inpatient duration. For the first time, plasma CRP level is demonstrated to assist for discerning patients with moderate to severe COVID-19 pneumonia from those with mild condition. This suggest CRP testing may be useful as an earlier indicator for severe illness and help physicians to stratify patients for intense care unit transfer.

## Data Availability

All datasets supporting these findings could be found in supporting sheet.
